# Exploring Biological Motion Processing in Parkinson’s Disease Using Temporal Dilation

**DOI:** 10.1371/journal.pone.0138502

**Published:** 2015-09-18

**Authors:** Ruihua Cao, Xing Ye, Xingui Chen, Long Zhang, Xianwen Chen, Yanghua Tian, Panpan Hu, Kai Wang

**Affiliations:** 1 Department of Geriatric Medicine, Anhui Provincial Hospital, Hefei, Anhui Province, China; 2 Department of Neurology, the First Affiliated Hospital of Anhui Medical University, Hefei, Anhui Province, China; University of Tuebingen Medical School, GERMANY

## Abstract

Biological motion (BM) perception is the compelling ability of the visual system to perceive complex animated movements effortlessly and promptly. A recent study has shown that BM can automatically lengthen perceived temporal duration independent of global configuration. The present study aimed mainly to investigate this temporal dilation effect of BM signals in Parkinson’s disease (PD) patients. We used the temporal dilation effect as an implicit measure of visual processing of BM. In all, 32 PD patients (under off-therapy conditions) and 32 healthy controls (HCs) participated in our study. In each trial, an upright BM sequence and an inverted BM sequence were presented within an interval in the center of the screen. We tested both canonical and scrambled BM sequences; the scrambled ones were generated by disturbing the global configuration of the canonical ones but preserving exactly the same local motion components. Observers were required to make a verbal two-alternative forced choice response to indicate which interval (the first or the second) appeared longer. Statistical analyses were conducted on the points of subjective equality (PSEs). We found that the temporal dilation effect was significantly reduced for PD patients compared with HCs in both canonical and scrambled BM conditions. Moreover, no temporal dilation effects of scrambled BM were shown in both early- and late-stage PD patients, while the temporal dilation effect of canonical BM was relatively preserved in the early stages.

## Introduction

Johansson (1973) first demonstrated the phenomenon of biological motion (BM) perception [1]. He showed that a set of twelve moving light points attached to the joints of the body sufficed to create a rich perception of a moving human figure. However, when the point-light displays are presented upside down, adequate perception is strongly impaired in a manner called the “inversion effect” [2–4]. BM provides socially relevant information that includes the identity of the moving stimulus, his or her actions, intentions, and even emotions. The human visual system is fine-tuned to detect and extract socially relevant information from BM rapidly and effortlessly. It has been suggested that performance on BM tasks may serve a hallmark of social cognition [5]. Therefore, a deficit in BM processing may have wide-ranging consequences for social perception and interpersonal functioning.

BM links the perception of local motion (carried by the motion of those points over time) with the perception of global form (carried by the position of points on the body), which are two qualities that involve largely different cortical processing streams [6]. The views on contributions of form and motion to the vivid perception of point-light displays have been controversial. While some studies claim that global form cues are critical, others emphasize the role of local motion signals [7–9].

Recently, a study claimed that BM could automatically lengthen perceived temporal duration independent of global configuration in healthy adults [10]. In this study, the authors adopted a duration discrimination paradigm and found that an upright BM sequence was perceived significantly longer than its inverted counterpart of the same physical duration. This temporal dilation could be extended to spatially scrambled biological sequences that shared the same local motion components as the canonical ones but without the gestalt of a global figure, which suggests that local BM is sufficient to trigger a special mechanism underlying temporal encoding of human motion.

The special mechanism remains unclear. However, the neural energy hypothesis has suggested that the perceived duration is a signature of the amount of neural energy required to represent a stimulus [11], which is in agreement with the findings that neural substrates associated with BM processing are at least partly different from those processing inverted stimuli. Neuroimaging studies have established the view that the posterior superior temporal sulcus (pSTS) plays a key role in processing BM [12–15]. Other regions involved include the fusiform body area (FBA) in the lateral fusiform gyrus and the extrastriate body area (EBA) in the occipital cortex [16–17].

Bradykinesia and motor slowness of Parkinson’s disease (PD) patients have been at the forefront of shaping the hypothesis that the inability to recognize the actions of others, a BM processing deficit, occurs in PD [18]. Moreover, PD is not just a motor disorder. Cognitive impairment, including social cognition, is frequent even in early-stage PD without dementia [19]. Much work has been done on the neurophysiological changes that take place in the brain. As PD is characterized by depletion of dopaminergic input from the substantia nigra to the striatum [20], therefore PD offers an ideal model to explore whether the basal ganglia and dopaminergic transmitter system are also related to the mechanisms underlying BM processing.

The dopamine depletion is clearly related to dysfunction of prefrontal cognitive areas through three different frontostriatal circuits: the dorsolateral circuit, the orbital circuit, and the anterior cingulate circuit [21]. In the early stages of PD, dopamine depletion largely affects the most dorsolateral portion of the head of the caudate nucleus in the dorsolateral frontostriatal circuit, largely preserving the processes based on the orbital frontostriatal circuit. With the progression of PD, the prefrontal cortex is directly affected by the neuropathology [22]; dopamine depletion within the striatum also affects the orbital frontostriatal circuit producing related dysfunction.

In the present study, we aimed to explore BM processing in PD patients. We investigated the temporal dilation effect of visual processing of BM signals in PD patients, which is a novel approach to studying BM processing. Although time perception per se may be impaired in PD patients, cognitive millisecond time processing is thought to be spared, which is the basis of our experimental design [23]. As BM signals automatically lengthen their perceived temporal duration, we can examine BM processing of observers by testing their temporal performance. We adopted 2 duration discrimination tasks to compare the temporal dilation effect of BM signals between PD patients and healthy controls (HCs). Experiment 1 adopted scrambled BM sequences (upright and inverted) and experiment 2 adopted canonical ones (upright and inverted). The inverted ones were used to trigger the temporal dilation effect, and the scrambled ones were representative of local BM. The present study aimed to compare BM processing in patients with PD and healthy participants. We also discuss possible underlying mechanisms related to the basal ganglia and dopaminergic transmitter system.

## Materials and Methods

### 2.1. Ethics statement

Written informed consent was obtained from all of the participants, and the study was conducted according to the principles expressed in the Declaration of Helsinki and was approved by the Ethics Committee of Anhui Medical University.

### 2.2. Participants

The participants in this study were 32 right-handed patients (as determined by scores of the Chinese hand preference questionnaire, which was revised according to the Edinburgh handedness test) affected by idiopathic PD, as well as 32 right-handed HCs of comparable age and education. An expert neurologist diagnosed the PD patients according to the London Brain Bank Criteria [24].

Exclusion criteria for PD patients were as follows: (1) patients who presented with a history of other neurological or psychiatric illnesses such as depression, cerebral infarction, or migraine, (2) dementia based on clinical examination or a Mini Mental State Examination (MMSE) score ≤ 24 [25], (3) the use of active central nervous system therapies other than levodopa and dopamine agonists, alcohol, or other substance abuse or dependence, and (4) deficits in seeing and hearing. PD patients were untreated or treated. Those treated had not taken medicine other than levodopa, and they participated in the experiment during an off-therapy condition, withdrawing pharmacological therapy no later than the evening before [26–27]. Table 1 shows demographic and clinical characteristics.

**Table 1:** Demographic Data, Clinical characteristics and Neuropsychological Findings of PD and HCs (Mean ± Standard Deviation).

	PD	HCs	*t*	*P* ^a^
Number	32	32	-	-
Age (years)	60.47±8.99	59.94±9.27	0.233	0.817
Gender (M/F)	18/14	20/12	0.773	0.442
Education Background (year)	7.13±4.50	7.63±3.61	0.490	0.626
Disease duration (years)	2.356±1.917	-	-	-
onset side (left/right)	12/20	-	-	-
Hoehn and Yahr stage				
Stage 1	9	-	-	-
Stage 2	11	-	-	-
Stage 3	11	-	-	-
Stage 4	1	-	-	-
Therapy conditon (treated/untreated)	13/19	-	-	-
Levodopa equivalent daily dose (mg/day)	0.313±0.167	-	-	-
MMSE score (out of 30)	27.22±1.88	27.59±1.78	0.821	0.415
VFT^b^	11.63±2.30	12.91±2.19	2.284	0.026
DS(f)^b^ (out of 8)	5.72±1.11	6.25±0.95	2.052	0.044
DS(b)^b^ (out of 7)	3.59±0.88	4.28±0.89	3.119	0.003

PD = Parkinson’s Disease; HCs = Healthy controls; DS (b) = Digital Span (backward); VFT = verbal fluency task; DS (f) = Digital Span (forward); MMSE = mini-mental state examination.

^a^ Analyzed by two-sided independent-samples *t*-tests.

^b^ Indicates a significant effect of group (*P* < 0.05).

### 2.3. Neuropsychological background tests

The neuropsychological background tests used were the MMSE, digit span (DS), and verbal fluency test (VFT). DS and VFT are considered sensitive to frontal lobe dysfunction. The Hamilton depression scale (HAMD) was also administered to evaluate possible depression.

### 2.4. Stimuli

Stimuli were generated and displayed using MATLAB (Mathworks) and the Psychophysics Toolbox extension [28]. The canonical point-light BM videos were adopted from Vanrie and Verfaillie [29]. They were originally created using a motion capture system and then synthesized by computer programs. In the scrambled BM sequences, the starting positions of each point were randomly displaced from their veridical positions about a central axis within the region identical in size to the canonical BM sequences, and the local motion cues were preserved. Only the global configuration information was entirely disrupted, so that familiar limb sequences were more difficult to identify. Inverted BM counterparts (canonical and scrambled) were derived by vertically mirror-flipping all of the motion sequences.

### 2.5. Experimental procedure

**Fig 1:**
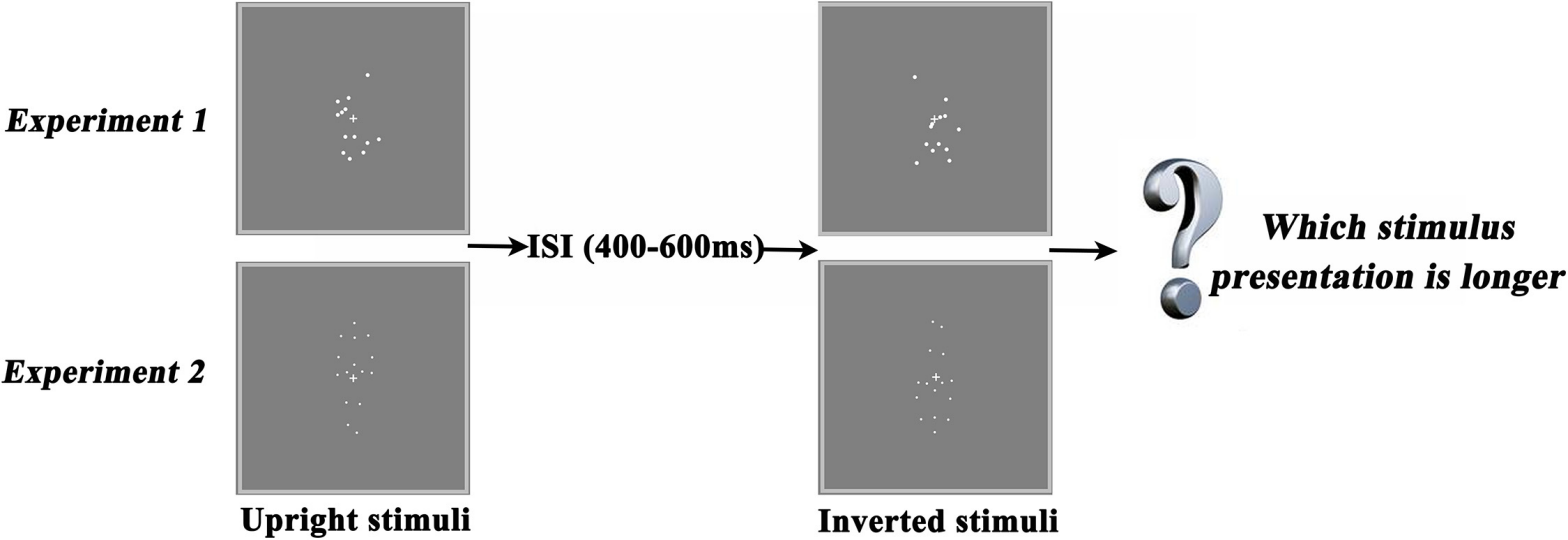
Task design. Experiment 1 employed scrambled biological motion (BM) sequences. Either an upright or an inverted scrambled BM stimulus was presented for an interval, followed by an interstimulus interval (ISI) and then the other stimulus. Experiment 2 employed canonical BM sequences. The presentation order of the two stimuli was randomized across trials.

Experiment 1 adopted scrambled BM sequences (upright and inverted) and experiment 2 adopted canonical ones (upright and inverted). Fig 1 represents the task used in our study. Stimuli were white on a gray background, and observers viewed them from approximately 80 cm away. In each trial, 2 stimuli (e.g., an upright canonical BM sequence and an inverted canonical BM sequence) were presented within an interval in the center of the screen, such that the dots subtended approximately 4.0° × 6.8° in visual angle. One of the stimuli (the upright or inverted figure) was randomly selected to be presented for 1000 ms; the other was displayed for 100 ms, 400 ms, 700 ms, 1000 ms, 1300 ms, 1600 ms, or 1900 ms, which resulted in a total of 7 test conditions. Thus, the difference between the presentation durations of the two stimuli (upright vs. inverted) was -900 ms, -600 ms, -300 ms, 0 ms, 300 ms, 600 ms, or 900 ms. To avoid a potential interference effect, a blank interval with a randomized duration of 400–600 ms was inserted between displays of the two stimuli. The presentation order of the two stimuli and the initial frame of the point-light display for each test stimulus were also randomized across trials (see S1 and S2 Video Clips).

Observers were required to make a verbal two-alternative forced choice to judge, as accurately as possible, which interval (the first or the second) was longer, regardless of what type of stimulus was shown. Participants were explicitly told not to count aloud or subvocally and that neither stimulus order nor content predicted stimulus presentation duration. The next trial started only after observers made their choice for the previous one.

To ensure that every observer was naive to the nature of the scrambled sequences, all observers were assigned to experiment 1 before experiment 2, since the canonical one may have introduced the concept of a human figure and had an impact on the subsequent scrambled one. After both experiments, we asked each subject what he or she could recognize from the sequences. Each experiment consisted of 70 trials, with 10 trials for each test condition. Thus, each observer performed 140 trials. A rest break was provided after every 20–30 trials.

### 2.6. Data analysis

**Fig 2:**
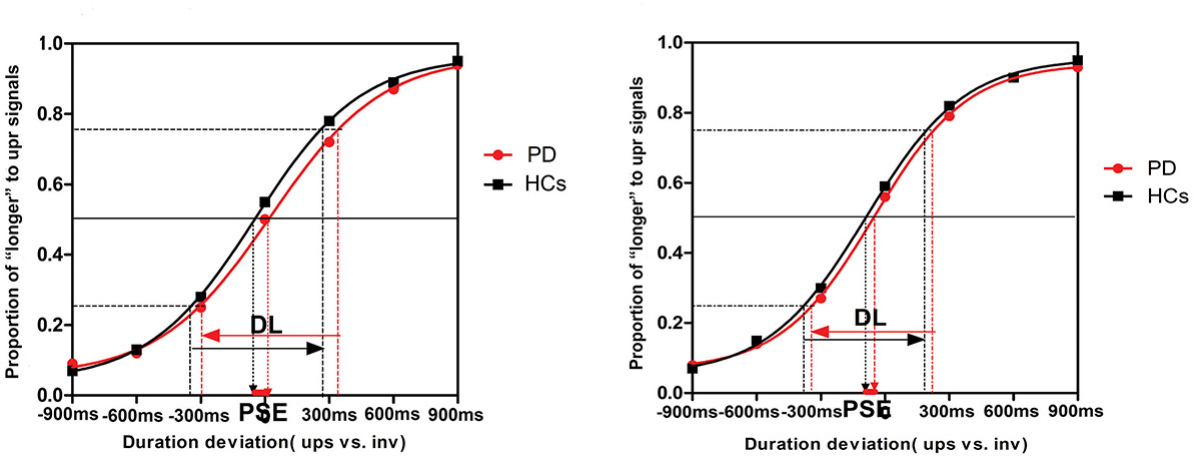
Psychometric functions in two conditions. (A) Psychometric function in the scrambled biological condition: the X-axis shows the deviation in the durations of the two stimuli (upright minus inverted), ranging from -900 ms to +900 ms; the Y-axis shows the proportion of “long” responses to upright stimuli. The arrow with the dashed line indicates the PSE, and the arrows with solid lines indicate the DL. A negative PSE indicates a temporal dilation effect for upright stimuli. (B) Psychometric function in the canonical biological condition. The psychometric functions shown represent the means of these individual functions.

The results of each individual observer from this two-alternative forced-choice task were fitted with a Boltzmann sigmoid function (Eq 1). Fig 2 depicts this function, in which the x-axis shows the difference between the presentation durations of the two stimuli (upright vs. inverted), ranging from -900 ms to +900 ms; the y-axis shows the proportion of “long” responses to upright stimuli. The statistical analyses were conducted on the point of subjective equality (PSE) and difference limen (DL). PSE referred to the point at which observers perceived the two stimuli equal in terms of the presentation duration, and it was estimated by the midpoint of the Boltzmann function:

f(x)=1/(1+exp[(x−x0)/ω]) (1)

A negative PSE indicated that when observers perceived two stimuli having as the same duration, the upright stimulus was presented for less time than the inverted counterpart in physical duration (i.e., temporal dilation); a positive PSE indicated the reverse (i.e., temporal compression). The DL was estimated by the interquartile range of the fitted function, and it was used to measure the temporal discrimination sensitivity [30].

The Shapiro-Wilk test was used to determine the normality of the data. The demographic and neuropsychological background data of the two groups were examined by independent-samples *t*-tests (two-sided). The one-sample *t*-test (two-sided) was used to detect whether the PSE was different from zero, and the Analysis of Variance (ANOVA) was used to detect overall main effects and interactions.

## Results

### 3.1. Demographic data, PD-related clinical characteristics, and neuropsychological findings

As analyzed by two-sided independent-samples *t*-tests, the demographic data, including age, gender, and educational levels, did not show significant differences between the two groups (*P* > 0.05) (see Table 1). A two-sided independent-samples *t*-test revealed that MMSE scores did not significantly differ between the two groups (*P* > 0.05); however, VFT and DS (DS [f] and DS [b]) scores were significantly different between the two groups (all *P* < 0.05) (see Table 1). Disease severity was assessed using the Hoehn–Yahr Scale (H&Y) [31], which is a commonly used system for describing the progression of PD symptoms and the relative levels of disability, including stages 1 to 5.

### 3.2. Duration discrimination task findings of 2 groups

Table 2 shows the PSEs and DLs for PD patients and HCs in the canonical and scrambled BM conditions. Fig 2 presents the fitted summary psychometric functions for the groups and tasks.

**Table 2:** Point of subjective equality (PSE) and difference limen (DL) for Parkinson’s disease (PD; n = 32) and control (n = 32) groups across canonical and scrambled BM conditions (Mean ± Standard Deviation)

Condition	Group	PSE	DL
Canonical BM	PD	-0.143±0.296*	2.178±1.319
	Control	-0.279±0.178	2.027±0.941
Scrambled BM	PD	0.008±0.193*	2.323±1.261
	Control	-0.123±0.193	2.064±0.920

* In comparison with healthy controls: analyzed by two-sided independent-samples *t*-tests, *P*< 0.05.

In the scrambled BM condition, all of the participants reported that they did not recognize any clue of a human figure when observing the stimuli, which included not only the inverted ones, but the upright ones as well. While in the canonical BM condition, all of the participants reported that they recognized walking human figures (upright or inverted) when observing the stimuli.

For HCs, a two-sided one-sample *t*-test revealed a significant negative PSE in the scrambled BM condition [*t*(31) = 3.619, *P* = 0.001] and in the canonical BM condition [*t*(31) = 8.842, *P* = 0.000], which suggests that BM signals lengthen their perceived temporal duration independent of global form (as demonstrated by Wang and Jiang [2012]).

**Fig 3:**
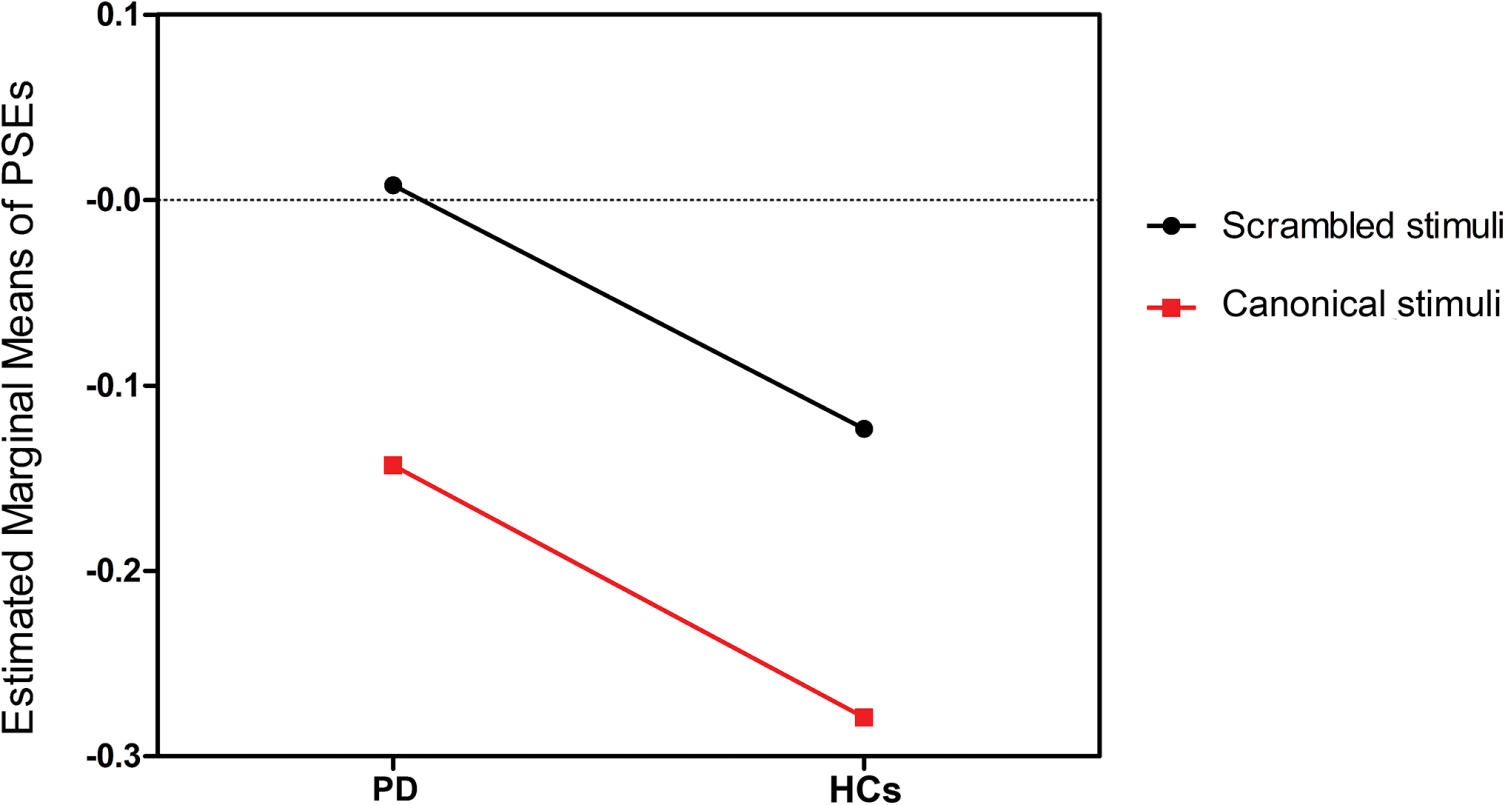
Estimated marginal means of points of subjective equality (PSEs) in experiments 1 and 2. Effects of group [Parkinson’s disease (PD) patients vs. health controls (HCs)] and task [canonical vs. scrambled biological motion (BM) condition] on PSEs and their interactions.

For PD patients, there was no significant negative PSE [*t*(31) = 0.232, *P* = 0.818] in the scrambled BM condition, suggesting that local BM processing may be impaired in PD patients. In the canonical BM condition, PD patients perceived upright BM sequences as significantly longer than its inverted counterpart [*t*(31) = 2.731, *P* = 0.010]. However, when compared with HCs, we found a significant difference between the two groups [*t*(62) = 2.223, *P* = 0.031], which indicates that such a temporal dilation effect of the upright BM sequences is preserved but scaled down. A two-way mixed ANOVA with group (PD patients vs. HCs) as between factor and task (canonical vs. scrambled BM condition) as within factor was performed on the PSEs. Analysis revealed significant main effects of group [*F*(1, 62) = 11.780, *P* = 0.001] and task [*F*(1, 62) = 15.481, *P* = 0.000]. There was no task × group interaction [*F*(1, 62) = 0.003, *P* = 0.954] (see Fig 3).

**Fig 4:**
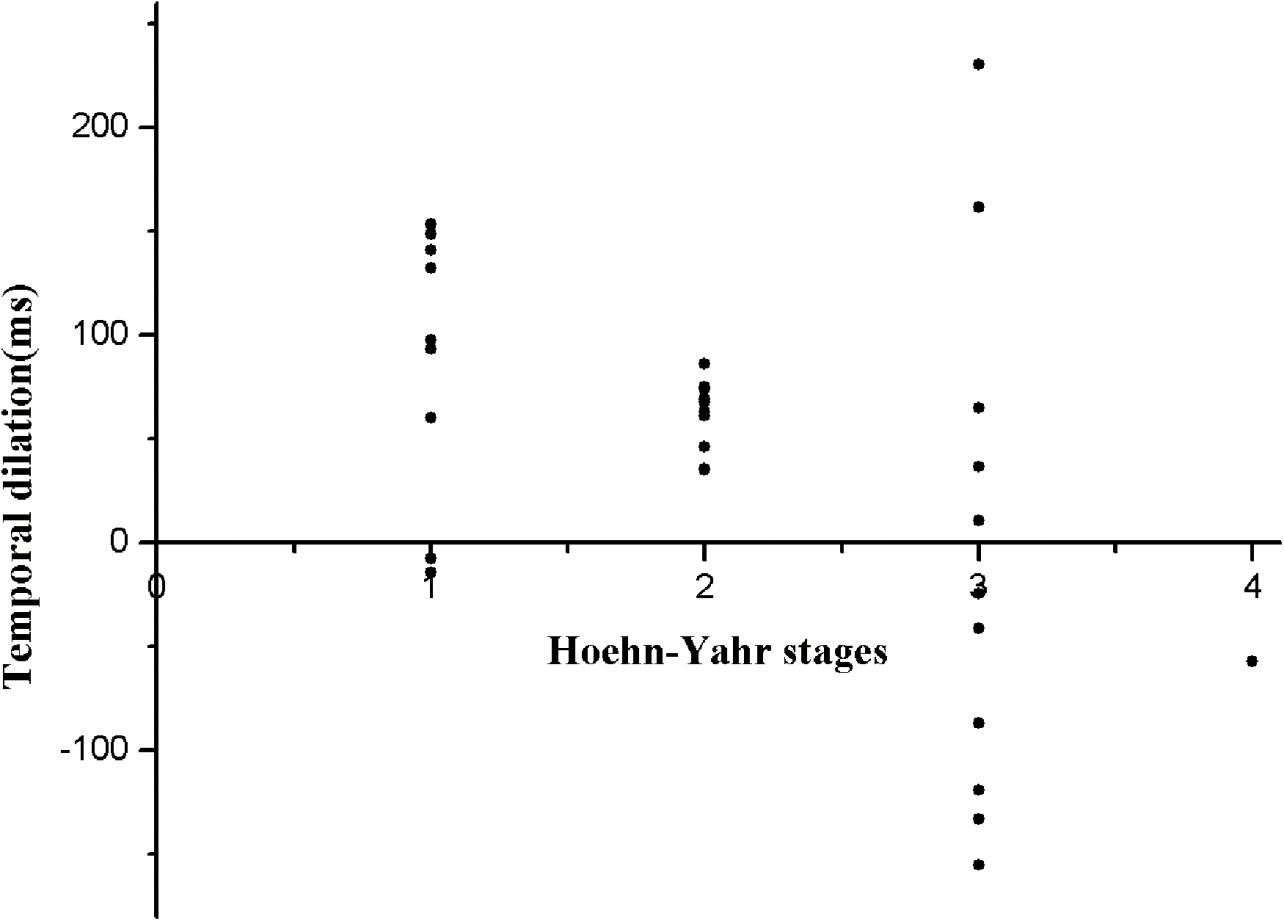
Group scatter plot of the distribution of the temporal dilation effect at different Hoehn-Yahr stages of Parkinson’s disease (PD) patients.

In addition, we found that PSEs in the canonical BM condition and Hoehn–Yahr stages were significantly correlated for PD patients (*r* = 0.495, *P* = 0.004) (see Fig 4). We further divided PD patients into 2 subgroups according to their Hoehn-Yahr stages; stage 1–2 (20 patients) belonged to early-stage PD and stage 3–4 (12 patients) belonged to late-stage PD.

For late-stage PD patients, results showed that the PSEs were not different from zero in both canonical and scrambled BM conditions [*t*(11) = 0.274, *P* = 0.789; *t*(11) = 0.372, *P* = 0.717, respectively], indicating deficits in BM processing. For early-stage PD patients, two-sided one-sample *t*-tests revealed a significant negative PSE for the canonical BM condition [*t*(19) = 7.273, *P* = 0.000001], but not for the scrambled BM condition [*t*(19) = 0.034, *P* = 0.973]. In addition, no significant differences were found between HCs and early-stage PD patients [*t*(50) = 0.653, *P* = 0.517], which suggests that scrambled BM processing was impaired and canonical BM processing was relatively preserved early in the course of PD.

**Fig 5:**
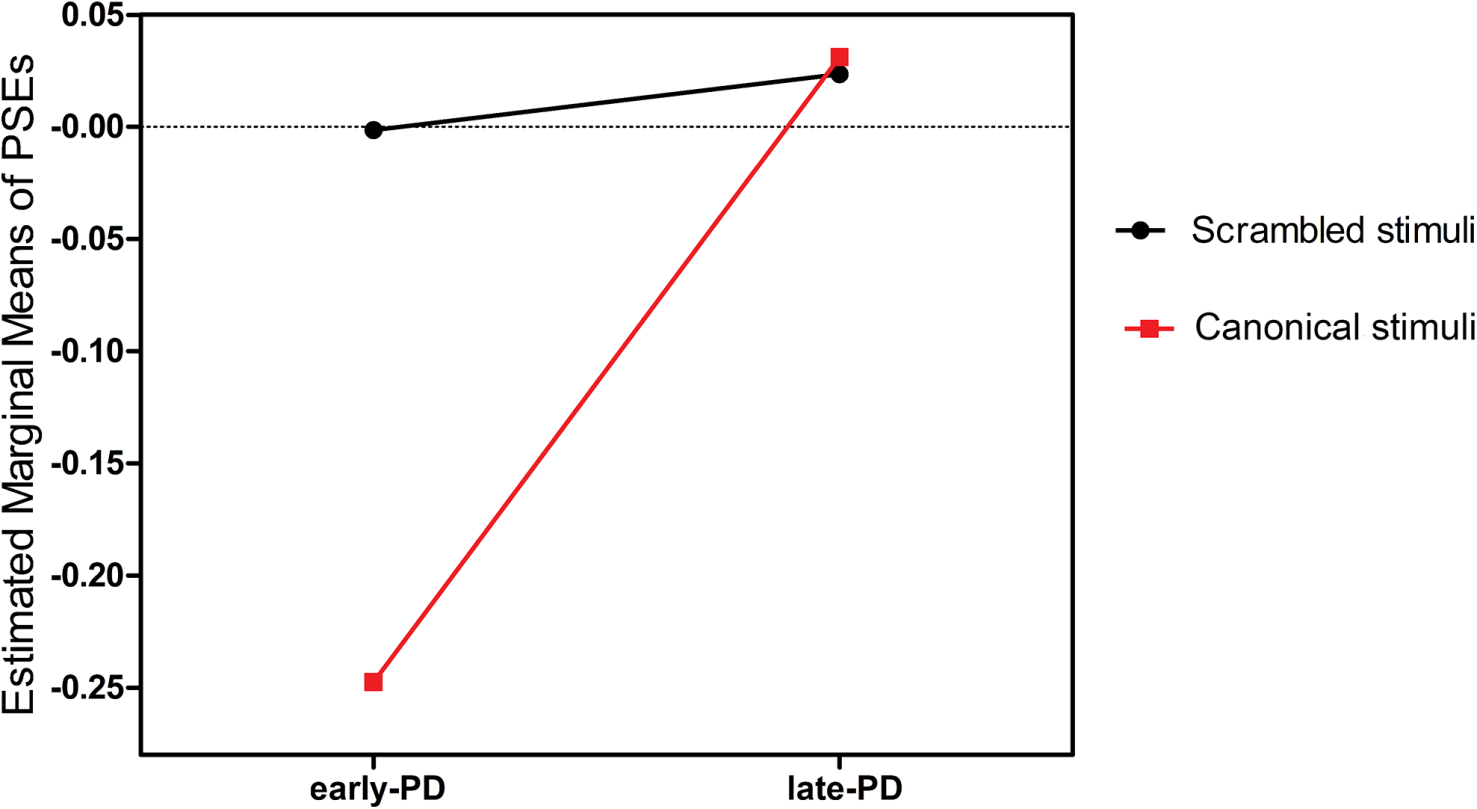
Estimated marginal means of points of subjective equality (PSEs) for Parkinson’s disease (PD) patients in experiments 1 and 2. Effects of subgroup [early-stage vs. late-stage PD] and task [canonical vs. scrambled biological motion (BM) condition] on PSEs and their interactions.

Another two-way mixed ANOVA with subgroup (early- vs. late-stage PD) as between factor and task (canonical vs. scrambled BM condition) as within factor was carried out on the PSEs for PD patients. The analysis showed significant main effects of subgroups [*F*(1, 30) = 6.300, *P* = 0.015], and a significant task × group interaction [*F*(1, 30) = 4.407, *P* = 0.040] (see Fig 5).

A two-sided independent-samples *t* test revealed that the differences between early-stage PD patients and late-stage PD patients were significant in the canonical BM condition [*t*(30) = 2.858, *P* = 0.008], and no differences were found between the two subgroups in the scrambled BM condition [*t*(30) = 0.347, *P* = 0.731].

A task × onset sides (sides of motor disorders at disease onset) ANOVA showed no significant main effect of onset sides [*F*(1, 30) = 0.186, *P* = 0.668]. A similar ANOVA failed to find a significant difference between medicated and non-medicated patients [*F*(1, 30) = 0.003, *P* = 0.955]. No differences were found between males and females in experiments 1 and 2 [*F*(1, 62) = 0.988, *P* = 0.324; *F*(1, 62) = 1.652, *P* = 0.204, respectively]. Temporal discrimination sensitivities (i.e., DL) of the observers were analyzed with a task × group ANOVA. There was no effect of task and group [*F*(1, 62) = 0.209, *P* = 0.649; *F*(1, 62) = 1.070, *P* = 0.303, respectively].

**Fig 6:**
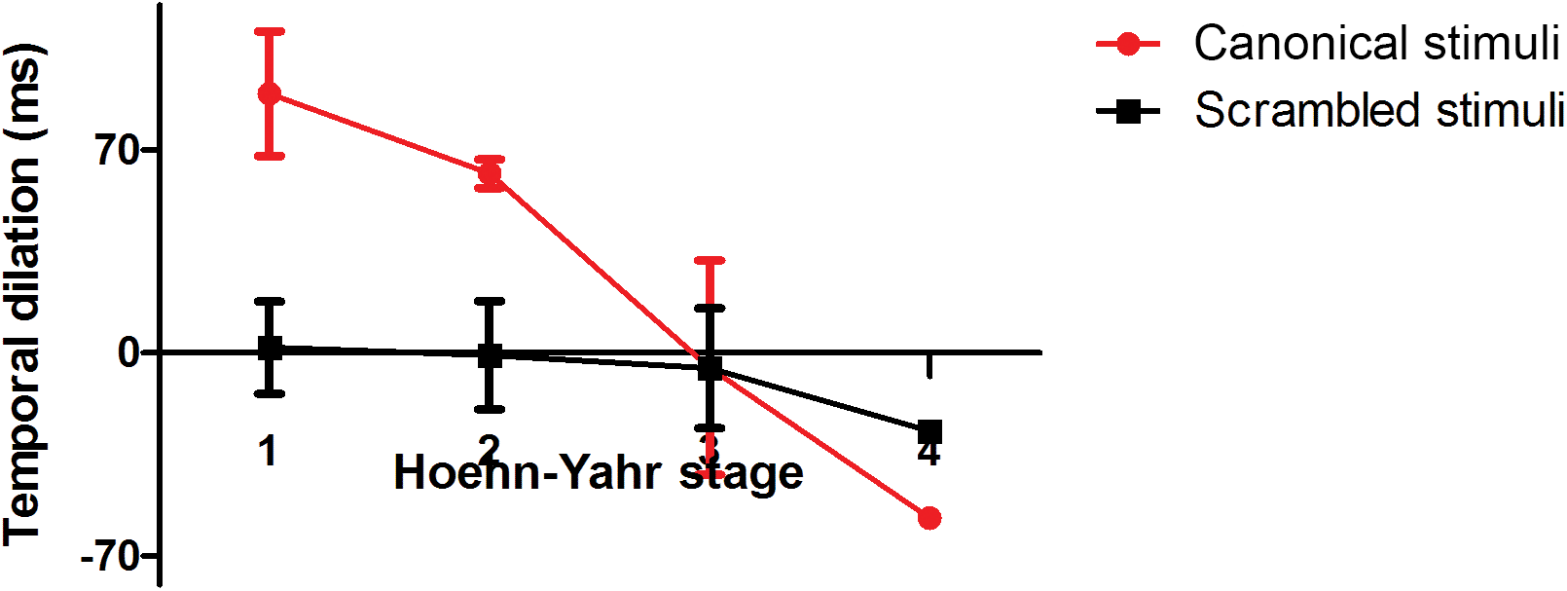
Decreases of the temporal dilation effect with disease stage increasing in canonical BM and scrambled BM conditions. Deficits in the temporal dilation effect in the canonical BM condition correlated with Hoehn-Yahr stages; patients in stages 3–4 were most involved, while deficits in the scrambled BM condition happened much earlier, independent of disease severity. Error bars indicate standard error.

Fig 6 shows decreases of the temporal dilation effect with disease stage increasing in canonical BM and scrambled BM conditions.

## Discussion

This study compared BM processing between patients with PD and healthy participants. A negative PSE indicated temporal dilation, and we used this effect as an implicit measure of visual processing of BM. Therefore, differences in PSE measures between PD patients and HCs may suggest a BM processing deficit in PD patients. We studied both canonical and scrambled BM sequences.

We found that the temporal dilation effect was significantly reduced for PD patients compared with HCs in both canonical and scrambled BM conditions. In the early stages of the disease, the effect was relatively preserved in the canonical BM condition. However, given that the temporal dilation effect in the canonical BM condition was significantly correlated with Hoehn-Yahr stages, we believe that impaired temporal performance may occur earlier than the data suggests since the sample size was limited.

The reduced temporal dilation effect of visual processing of BM signals agrees with a previous study that demonstrated impairments in perceptual sensitivity to kinematic invariants in PD. In this study, the invariant properties characterizing motion perception in neurologically healthy individuals (the “two-thirds power law”) lost their constraints on motion perception in PD patients [32].

As PD is characterized by tremor, rigidity, bradykinesia, and postural abnormalities, the reduced temporal dilation effect of BM processing found in our study supports coupling between action and perception. Ample evidence suggests action-perception coupling in humans and nonhuman primates [33–38]. One possible mechanism is called “mirror neuron system” (MNS); a group of neurons that were originally discovered in the premotor cortex of monkeys' discharges when a movement is executed and when the same movement is observed [39]. Previous studies have shown that action observation can have a positive additional impact on rehabilitation of motor deficits after stroke by activating motor areas containing MNS [40–42]. Two recent LFP studies in PD patients suggested that basal ganglia might be engaged by activity of the human MNS [43–44], which may contribute to impaired temporal performance in PD patients. Pavlova et al. also proposed that there might be an inherent brain network responsible for the coupling; BM perception does not necessarily positively correlate with motor ability or experience per se [45].

In our study, the temporal dilation effect in the scrambled BM condition was impaired early in the course of the disease, while the effect in the canonical BM condition was relatively preserved in the early stages. Thus, the different patterns of impairment may indicate different mechanisms underlying local and global BM processing, which agrees with the theoretical model by Giese and Poggio [46]. Observed differences between canonical and scrambled BM processing are in accordance with previous fMRI studies [47–50]. Downing et al. observed a stronger activation for canonical BM displays in the EBA than for scrambled controls with identical motion signals [47]. In addition, Grossman and Blake found a slight increase in activation for BM over the scrambled control in the EBA and a strong and significant difference in the STS [48].

Underlying pathophysiological mechanisms may include deficits due to dopamine depletion in frontostriatal circuits in PD patients. Evidence suggests that dopamine depletion is involved in dynamic visuospatial processing [43, 51–52]. Deficits in DS and VFT found in our study indicated frontal lobe dysfunction. Moreover, the progressive pathophysiological changes that occur in frontostriatal circuits in PD patients may account for the successive damage of temporal dilation effects in scrambled and canonical BM conditions. In our study, only the “off” levodopa condition was completed. Further studies are needed to compare both “on” and “off” levodopa conditions to establish the influence of dopaminergic involvement.

In our study, we investigated visual processing of BM signals in PD patients using the temporal dilation effect. However, time perception per se may be impaired in PD patients. Previous findings have suggested that the cerebellum may contribute to the precise timing of salient events in the millisecond range, which determines the duration of a stimulus independently from basal ganglia activity or the onset and end of movements [53–55]. Findings from recent studies [23,56] agree with the observation that PD patients are not impaired in cognitive millisecond time processing. The cerebellum is also thought to be engaged in the neural network dedicated to visual processing of body motion through a structural pathway between the right posterior STS and the left cerebellum [57]. Increasing evidence suggests that the cerebellum participates in the pathophysiology of PD; major roles may include pathological and compensatory effects [58–59].

The temporal dilation effect is a novel approach to study BM processing, but it is not precise and evidence is still limited. In the study by Wang and Jiang (2012), an additional control experimental condition with “critical biological characteristics removed” was employed to determine whether differences in temporal processing found between groups were specific to BM. The absence of a truly non-BM control condition represents the major limitation of our study, which prevents us from drawing firm conclusions concerning BM processing deficits in PD patients. Further research in this area is required.

## Conclusion

In summary, the temporal dilation effect was significantly reduced in PD patients compared with HCs, which may indicate deficits in BM processing.

## Supporting Information

S1 File Supplementary data. Raw data from the present study.
(XLS)

S1 Video Clip BioMotion. This video clip presents the canonical biological motion sequences used in experiment 2.
(GIF)

S2 Video Clip BioMotionScramble. This video clip presents the scrambled biological motion sequences used in experiment 1.
(GIF)
