# Correction: Exploring Biological Motion Processing in Parkinson's Disease Using Temporal Dilation

**DOI:** 10.1371/journal.pone.0140517

**Published:** 2015-10-08

**Authors:** The PLOS ONE Staff

## Notice of Republication

This article was republished on September 25, 2015, due to the release of confidential or copyrighted material. The publisher apologizes for the error. Please download this article again to view the correct version.
