# Supplementary figures and images for: Exploring Biological Motion Processing in Parkinson’s Disease Using Temporal Dilation
Source: PLoS One. 2015 Sep 18;10(9):e0138502. doi: 10.1371/journal.pone.0138502 (PMC4575113; doi:10.1371/journal.pone.0138502)

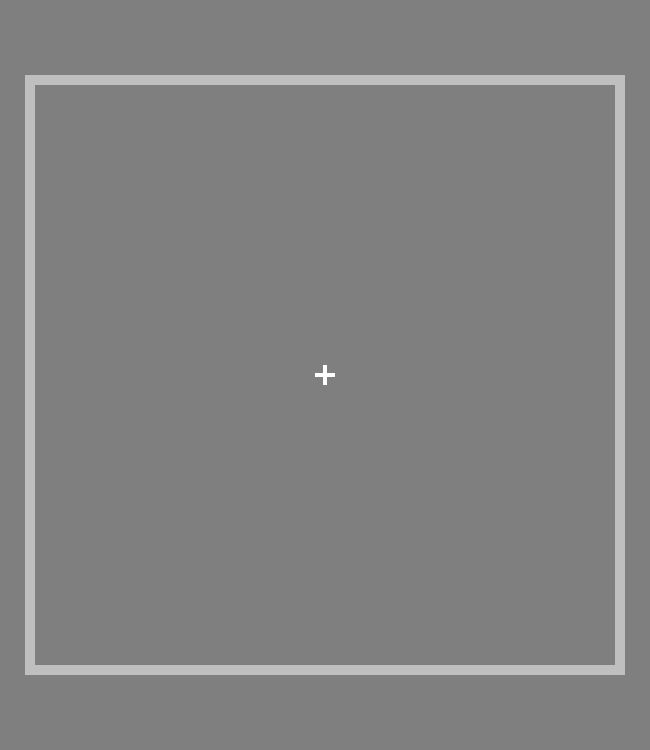

Supplement: S1 Video Clip — This video clip presents the canonical biological motion sequences used in experiment 2. (GIF) [file pone.0138502.s002.gif]

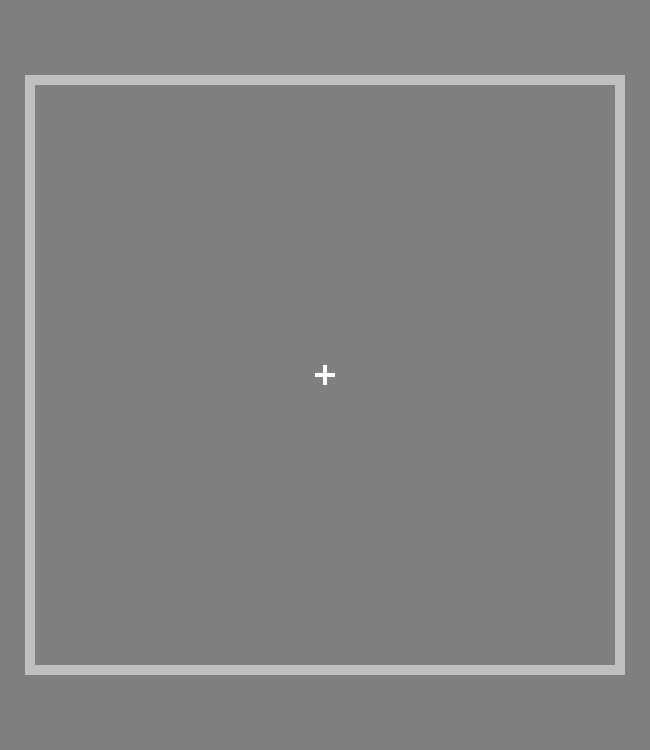

Supplement: S2 Video Clip — This video clip presents the scrambled biological motion sequences used in experiment 1. (GIF) [file pone.0138502.s003.gif]
